# Cardiometabolic 2.0: Redefining Cardiovascular Prevention Through SGLT-2 Inhibitors and GLP-1 Receptor Agonists

**DOI:** 10.3390/life16050756

**Published:** 2026-05-01

**Authors:** Maria-Daniela Tanasescu, Andrei-Mihnea Rosu, Alexandru Minca, Maria-Mihaela Grigorie, Delia Timofte, Dorin Ionescu

**Affiliations:** 1 Department of Semiology-Emergency, University Hospital, Carol Davila University of Medicine and Pharmacy, 022328 Bucharest, Romania; maria.tanasescu@umfcd.ro (M.-D.T.); dorin.ionescu@umfcd.ro (D.I.); 2 Department of Cardiology, Prof. Dr. Agrippa Ionescu Emergency Hospital, 077015 Balotesti, Romania; andrei-mihnea.rosu@drd.umfcd.ro; 3 Department of Dentistry, Faculty of Dentistry, Carol Davila University of Medicine and Pharmacy, 020021 Bucharest, Romania; maria.grigorie@umfcd.ro; 4 Department of Dialysis, Bucharest Emergency University Hospital, 050098 Bucharest, Romania; delia.timofte@gmail.com

**Keywords:** SGLT-2 inhibitors, GLP-1 receptor agonists, cardiovascular prevention, cardiometabolic medicine, heart failure, atherosclerotic cardiovascular disease, obesity, chronic kidney disease, type 2 diabetes, cardiorenal–metabolic syndrome

## Abstract

Cardiometabolic disease is increasingly shaped by the overlap among obesity, type 2 diabetes, chronic kidney disease, heart failure, and atherosclerotic cardiovascular disease, underscoring the need for prevention strategies that extend beyond glucose-centered care. This narrative review critically examines the mechanistic rationale, clinical evidence, guideline evolution, and practical implementation of sodium-glucose cotransporter-2 inhibitors (SGLT-2 inhibitors) and glucagon-like peptide-1 receptor agonists (GLP-1 receptor agonists) within the cardiorenal–metabolic continuum. A structured literature search was conducted in PubMed, Scopus, and Web of Science, focusing primarily on publications from January 2019 to March 2026, with selected landmark studies from earlier years included for context. Priority was given to randomized controlled trials, major cardiovascular and kidney outcome trials, meta-analyses, clinical practice guidelines, scientific statements, and expert consensus documents. The reviewed evidence indicates that SGLT-2 inhibitors show the most consistent benefits in reducing heart failure events, slowing chronic kidney disease progression, and lowering cardiorenal risk, whereas GLP-1 receptor agonists are more strongly associated with reductions in major adverse cardiovascular events, residual atherosclerotic risk, and body weight. Emerging data also support extension of this therapeutic paradigm beyond diabetes, particularly in obesity-associated cardiovascular risk. Contemporary care is increasingly moving toward phenotype-informed treatment selection, earlier organ-protective intervention, and multidisciplinary management, although cost, access, tolerability, and implementation barriers remain important limitations. SGLT-2 inhibitors and GLP-1 receptor agonists are therefore central to modern cardiovascular prevention across the cardiovascular–kidney–metabolic spectrum. In this context, the proposed Cardiometabolic 2.0 framework may serve as a clinically oriented model for integrating these therapies within contemporary organ-protective care.

## 1. Introduction

Cardiometabolic disease is one of the major health challenges of the 21st century, driven by the rising prevalence of obesity, type 2 diabetes (T2D), and cardiovascular disease (CVD). In 2021, an estimated 529 million people were living with diabetes worldwide, with a global age-standardized prevalence of 6.1%, and projections suggest that this number may rise to more than 1.31 billion by 2050 [1]. CVD remains the leading cause of death and disability worldwide, while the burden of excess adiposity continues to increase. From 1990 to 2021, global deaths and disability-adjusted life-years (DALYs) attributable to high body mass index (BMI) increased by more than 2.5-fold, and in 2021 the main causes of high-BMI-attributable DALYs included diabetes mellitus, ischemic heart disease, hypertensive heart disease, chronic kidney disease (CKD), and stroke [2,3]. In the same year, 52.2% of global T2D DALYs were attributable to high BMI, supporting the view that obesity is a major upstream driver of cardiometabolic disease [1,3].

Although the link between hyperglycaemia and diabetic complications is well established, a glucose-centered approach to T2D management does not adequately address the broader burden of cardiovascular and renal disease. Intensive glycaemic control reduces microvascular complications, but its effect on major cardiovascular (CV) outcomes has been limited, and in some settings safety concerns have narrowed its overall clinical benefit [4,5]. Over the past decade, large CV outcome trials have changed the treatment landscape by showing that some glucose-lowering therapies, particularly sodium-glucose cotransporter-2 inhibitors (SGLT-2 inhibitors) and glucagon-like peptide-1 receptor agonists (GLP-1 receptor agonists), provide CV and renal benefits that are only partly explained by glucose lowering [4,5,6]. Current recommendations now support the use of these agents according to cardiovascular, renal, and metabolic risk, rather than glycated haemoglobin (HbA1c) alone [6].

This change reflects a broader shift in how cardiometabolic disease is understood. Obesity, T2D, CKD, heart failure (HF), and atherosclerotic cardiovascular disease (ASCVD) rarely occur in isolation. They cluster through shared mechanisms that include insulin resistance, chronic inflammation, endothelial dysfunction, adipose tissue dysfunction, and neurohormonal activation. As a result, treatment is moving away from a strictly glucocentric model and toward one centered on organ protection, residual risk reduction, and earlier intervention across overlapping phenotypes [7].

Within this framework, SGLT-2 inhibitors and GLP-1 receptor agonists have become central therapies in contemporary cardiometabolic prevention, although their clinical profiles differ. SGLT-2 inhibitors are more consistently associated with reductions in hospitalization for heart failure, progression of CKD, and cardiorenal events, whereas GLP-1 receptor agonists are more closely aligned with reductions in major adverse CV events, body weight, and residual atherosclerotic risk [8,9]. At the same time, these distinctions should not be interpreted as absolute therapeutic separation. Many patients present with overlapping phenotypes, and the relative positioning of each class depends not only on trial signals, but also on guideline context, kidney function, comorbidity burden, treatment goals, and the strength of evidence available for each outcome domain More recent evidence has extended this therapeutic paradigm beyond diabetes, showing that pharmacological treatment of obesity can reduce major adverse CV events in people with overweight or obesity and established CVD even in the absence of diabetes [10].

In this review, the term “Cardiometabolic 2.0” is used as a practical interpretive framework to describe this transition from glucose-centered management to integrated, phenotype-informed, organ-protective prevention across the cardiovascular–kidney–metabolic continuum. As used here, the term does not propose a new disease classification, a formal staging system, or a guideline-endorsed treatment algorithm. Rather, it is intended as a didactic and integrative model that helps translate existing cardiovascular–kidney–metabolic concepts into a therapeutic perspective centered on residual risk domains, organ vulnerability, and clinically dominant phenotype. It is not intended to replace existing CKM or cardiorenal–metabolic frameworks, but to highlight their therapeutic implications for clinical decision-making. Accordingly, the purpose of this review is not to establish a novel paradigm, but to provide a structured narrative synthesis of how contemporary mechanistic evidence, outcome trials, and multidisciplinary recommendations collectively support a broader, more phenotype-aware approach to cardiovascular prevention. The aim of this narrative review is to examine the mechanistic rationale, clinical trial evidence, guideline evolution, and practical implementation of SGLT-2 inhibitors and GLP-1 receptor agonists in CV prevention, and to discuss how these agents may be positioned within an integrated cardiorenal–metabolic care model. Throughout the review, this positioning is presented as an interpretive clinical synthesis grounded primarily in randomized trial data and guideline documents, while recognizing that some areas remain hypothesis-generating, indirect, or insufficiently resolved for prescriptive treatment hierarchies.

## 2. Narrative Review Design and Literature Search Strategy

This manuscript was developed as a narrative review to synthesize and critically contextualize the contemporary role of SGLT-2 inhibitors and GLP-1 receptor agonists in cardiovascular prevention within the broader cardiorenal–metabolic continuum. The objective was not to perform a formal quantitative evidence synthesis, but rather to provide a clinically oriented and conceptually integrative overview of a rapidly evolving field. From the outset, the review was conceived as an interpretive and practice-oriented synthesis intended to connect mechanistic evidence, cardiovascular and kidney outcome data, and contemporary guideline positioning, rather than as a systematic review designed to generate pooled effect estimates or a comprehensive study inventory.

To inform this review, a structured literature search was conducted in PubMed, Scopus, and Web of Science. The primary search window covered publications from January 2019 to March 2026; selected landmark articles published before 2019 were included when considered essential to understanding the development of cardiovascular outcomes-based therapy and the emergence of cardiorenal–metabolic treatment paradigms. Earlier studies were therefore retained selectively when they represented foundational cardiovascular outcome trials, seminal mechanistic observations, or major turning points in guideline evolution.

The search strategy combined controlled vocabulary and free-text terms related to the two therapeutic classes and their principal clinical domains. Representative search terms included: “SGLT-2 inhibitors,” “GLP-1 receptor agonists,” “cardiovascular prevention,” “cardiometabolic,” “cardiorenal–metabolic,” “atherosclerotic cardiovascular disease,” “heart failure,” “chronic kidney disease,” “obesity,” “type 2 diabetes,” “cardiovascular outcome trials,” and “guidelines.” Search terms were used alone and in combination, and the reference lists of relevant articles were also screened to identify additional key publications. Boolean combinations were adapted to each database and generally linked intervention terms with major disease domains and evidence categories (e.g., cardiovascular outcomes, heart failure, chronic kidney disease, obesity, mechanisms, guidelines, and consensus statements). A detailed summary of the search framework, including databases, time window, representative Boolean logic, thematic domains, and exclusion principles, is provided in Supplementary Table S1 to improve transparency and reproducibility within the limits of a narrative review design.

Priority was given to randomized controlled trials, particularly major cardiovascular, heart failure, and kidney outcome trials; meta-analyses; evidence-based clinical practice guidelines; scientific statements; and expert consensus documents from relevant cardiology, diabetology, nephrology, and obesity medicine societies. Seminal mechanistic and translational studies, as well as selected high-quality narrative and state-of-the-art reviews, were also examined to support interpretation of biological pathways and therapeutic positioning. Study selection was guided by clinical relevance, methodological robustness, recency, and direct relevance to the manuscript’s central theme. When multiple publications addressed similar questions, priority was given to the most definitive, contemporary, and clinically influential sources. In practical terms, evidence prioritization followed a hierarchical logic: first, dedicated cardiovascular, heart failure, kidney, and obesity outcome trials; second, major meta-analyses and high-quality comparative syntheses; third, current international guidelines, scientific statements, and expert consensus documents; and fourth, mechanistic, translational, and imaging studies used to support biological plausibility and therapeutic interpretation. Narrative reviews were used selectively, mainly to contextualize areas in which primary evidence remained heterogeneous or conceptually fragmented.

Although this was not a systematic review, explicit selection boundaries were applied. Publications were generally excluded when they were clearly outside the review scope, duplicated the same trial population without adding materially relevant information, represented superseded interim analyses when final reports were available, or consisted of case reports, brief opinion pieces, or low-informative commentaries without direct relevance to the manuscript’s central questions. Preference was given to peer-reviewed English-language publications. Where multiple reports from the same evidence stream were available, the most mature, clinically informative, and methodologically robust source was preferentially cited.

Because phenotype-based cardiovascular prevention increasingly draws on multiple evidence layers, the review intentionally integrated randomized trials, meta-analyses, guideline documents, and mechanistic studies. However, these evidence types were not treated as equivalent. Randomized outcome data and formal guideline recommendations were used as the main basis for clinical positioning, whereas mechanistic and translational studies were used to explain biological plausibility, and observational or indirect comparative data were interpreted more cautiously and identified as hypothesis-supporting rather than definitive.

Because the aim of the present article was to provide an interpretive and practice-oriented synthesis, this review was conducted as a narrative review rather than a systematic review. Accordingly, no review protocol was prospectively registered, no formal risk-of-bias assessment tool was applied across included studies, and no pooled meta-analytic estimates were generated. This approach allows broader conceptual integration across trials, guidelines, and mechanistic literature, but it also carries an inherent risk of selection bias and should therefore be interpreted as a structured expert synthesis rather than an exhaustive systematic evidence appraisal. For the same reason, no PRISMA flow diagram is presented.

Finally, special attention was paid during revision to areas in which the evidence base is heterogeneous or potentially overstated in narrative discussion, including variability in major adverse cardiovascular event signals across trials, the less consistent heart failure profile of GLP-1 receptor agonists, the still-evolving interpretation of renal benefit beyond albuminuria-based endpoints, and the distinction between guideline-supported recommendations and author-derived clinical interpretation. These points were incorporated to improve balance, reduce overstatement, and better align the review’s conclusions with the strengths and limitations of the available data.

## 3. The Cardiometabolic 2.0 Concept

### 3.1. From Glucose Lowering to Risk Modification

For many years, the management of T2D was centered on glycaemic control, with HbA1c as the main therapeutic target and microvascular protection as the principal clinical goal. This approach improved outcomes such as retinopathy, nephropathy, and neuropathy, but had a much smaller effect on macrovascular events, HF, and diabetes-related mortality, making it clear that HbA1c reduction alone does not capture the full burden of risk in T2D. As a result, treatment goals have shifted toward a broader, outcome-driven model that gives greater priority to CV, renal, and metabolic prognosis [11,12,13].

This change reflects a growing recognition that stepwise intensification based mainly on glycaemic thresholds may postpone the use of therapies capable of preventing irreversible CV and renal injury. Current treatment strategies therefore place greater value on early risk reduction, with attention to comorbidity burden, hypoglycaemia risk, body weight, kidney function, and long-term cardiorenal outcomes. In this setting, SGLT-2 inhibitors have moved earlier in the treatment pathway because their benefits extend beyond glycaemic efficacy and include protection against CV and renal complications [13]. The central question in modern diabetes care is no longer how far HbA1c should be lowered, but which complications can be prevented and how the overall course of disease can be changed [14].

This shift has also changed how the relationship between diabetes and CVD is understood. Rather than parallel conditions, they are now better viewed as linked manifestations of a shared cardiometabolic process associated with persistent residual risk despite improvements in glucose, lipid, and blood pressure (BP) control [14]. Cardiometabolic prevention has therefore moved toward a more integrated and risk-based model that prioritizes organ protection, early identification of high-risk phenotypes, and multidisciplinary care.

This change is bigger than a simple expansion of treatment options within diabetology. It reflects a different way of thinking about prevention. In current practice, the more relevant question is often not how much a therapy lowers glucose, but whether it changes the course of HF, CKD, ASCVD, or obesity-related risk. In this review, the term Cardiometabolic 2.0 refers to that shift: away from surrogate metabolic targets and toward earlier, phenotype-informed prevention of multisystem clinical disease.

### 3.2. The Cardiorenal–Metabolic Continuum

The cardiorenal–metabolic continuum describes the close and progressive interaction among obesity, insulin resistance, T2D, CKD, and CVD. These conditions do not develop in isolation. They evolve through shared biological pathways and tend to accelerate one another once organ injury begins. In this framework, excess adiposity, dysglycaemia, hypertension, and dyslipidaemia are early manifestations of a broader disease process that may later lead to albuminuria, declining kidney function, vascular disease, HF, and premature death [15,16].

Several mechanisms drive this continuum. Chronic low-grade inflammation, oxidative stress, endothelial dysfunction, and activation of the renin–angiotensin–aldosterone and sympathetic nervous systems sustain a cycle of vascular, renal, and metabolic injury. In the kidney, these abnormalities promote glomerular hyperfiltration, albuminuria, sodium retention, fibrosis, and progressive loss of filtration capacity. At the same time, insulin resistance and adipose tissue dysfunction create a pro-inflammatory, pro-atherogenic environment, while CV dysfunction further impairs renal perfusion and metabolic stability [16,17]. Disease progression is therefore multidirectional: injury in one organ system amplifies dysfunction in the others.

This model has direct clinical relevance. A patient with central obesity and hypertension may already be on a trajectory toward CKD and CVD long before symptoms appear. Early reductions in estimated glomerular filtration rate (eGFR) or mild albuminuria may reflect a broader cardiometabolic risk state rather than an isolated renal abnormality. For this reason, the cardiorenal–metabolic continuum should be seen as a framework for early risk detection and earlier intervention. It supports routine assessment of obesity, BP, dysglycaemia, kidney function, and albuminuria, together with closer collaboration between cardiology, nephrology, and metabolic medicine.

Cardiometabolic 2.0 is not intended to replace established CKM or cardiorenal–metabolic models. Rather than proposing a distinct nosological entity or a new staging framework, the term is used here as an interpretive clinical lens through which the therapeutic implications of these established models may be viewed more explicitly. It is meant to build on them and bring their therapeutic meaning into sharper focus. In this sense, its purpose is didactic rather than classificatory: to emphasize how contemporary outcome trials and guideline evolution have increasingly shifted treatment selection toward dominant risk domains, organ vulnerability, and phenotype-informed prevention. More specifically, it reflects the way recent outcome trials have pushed treatment decisions toward dominant clinical phenotype and organ vulnerability, while still recognizing the considerable overlap between metabolic, renal, and cardiovascular disease. Accordingly, Cardiometabolic 2.0 should be understood in this review as a practical summary of therapeutic orientation within an already recognized cardiorenal–metabolic continuum, not as a replacement for existing conceptual frameworks and not as a prescriptive algorithm validated independently of them.

### 3.3. A New Preventive Framework in CV Medicine

CV prevention is now moving beyond the traditional control of single risk factors toward a multidimensional approach that recognizes the overlap among diabetes, obesity, CKD, HF, and ASCVD. Within this framework, prevention aims to slow or interrupt the progression of multisystem disease before irreversible CV or renal damage becomes established [7,11].

The rationale is both clinical and biological. Excess and dysfunctional adipose tissue often precede dysglycaemia, hypertension, dyslipidaemia, albuminuria, reduced kidney function, and subclinical CV injury, creating a long preclinical phase during which risk is rising but major events have not yet occurred [18]. Chronic inflammation, insulin resistance, endothelial dysfunction, adipose tissue dysregulation, and neurohormonal activation link these disorders across organ systems and help explain why HF and CKD are fundamental components of cardiometabolic disease rather than secondary complications [9].

This has major implications for prevention. Risk assessment now needs to reflect total CV burden, including HF and CKD, rather than focusing only on atherosclerotic events [18,19]. Prevention also needs to begin earlier, with attention to subclinical disease, kidney markers, metabolic dysfunction, and phenotype-specific risk. In practical terms, this supports coordinated use of lifestyle intervention, therapies with CV and renal benefit, and multidisciplinary care across cardiology, nephrology, diabetology, and primary care [7,18,20]. In this sense, Cardiometabolic 2.0 describes a preventive model that is earlier, broader, and more closely aligned with the full cardiorenal–metabolic burden carried by the patient. Established CKM frameworks describe disease clustering and progression; Cardiometabolic 2.0 is used here more narrowly, to emphasize the therapeutic translation of those established insights into phenotype-aware clinical reasoning. It should therefore be understood as a didactic framing device within the existing cardiovascular–kidney–metabolic paradigm, rather than as a separate conceptual system or a formal algorithm for treatment selection.

**Figure 1:**
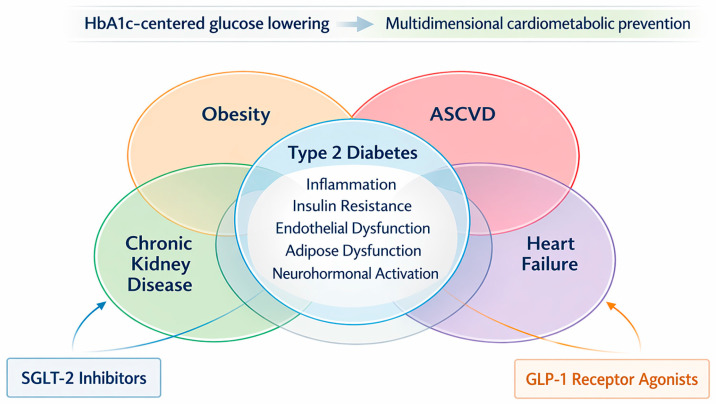
The Cardiometabolic 2.0 framework. The figure should be interpreted as a conceptual synthesis rather than as a formal staging system or guideline-endorsed algorithm.

To summarize these interrelated shifts in disease conceptualization, shared pathophysiology, and preventive strategy, Figure 1 illustrates the Cardiometabolic 2.0 framework as an integrated model linking metabolic, renal, and CV risk domains.

## 4. SGLT-2 Inhibitors: Mechanistic Basis for CV Protection

### 4.1. Glucose-Dependent and Glucose-Independent Effects

SGLT-2 inhibitors were developed as glucose-lowering agents that act in the proximal renal tubule by blocking glucose reabsorption and increasing urinary glucose excretion, an effect that is independent of insulin action [21]. Their clinical profile, however, extends well beyond glycemic control. Contemporary reviews note that these agents modify a broad set of CV risk pathways, including BP, weight, visceral adiposity, arterial stiffness, albuminuria, uric acid, oxidative stress, and renal sodium handling, which already places them outside the narrower framework of HbA1c-centred diabetes treatment [22,23]. Early separation of event curves in outcome trials further supports this view. Empagliflozin was associated with an early reduction in major CV and renal outcomes, with CV protection reported from the first months of therapy, a time course that is difficult to attribute to glucose lowering alone [21,23]. This interpretation is strengthened by post hoc EMPA-REG analyses showing that although empagliflozin reduced HbA1c variability, the reduction in CV death was not mediated by that change and was independent of glycaemic control during the trial [24].

These observations support the view that the cardiovascular effects of SGLT-2 inhibitors are only partly related to their antihyperglycaemic action. Their benefit appears to arise from a combination of hemodynamic, renal, metabolic, and myocardial effects that operate in parallel and may become clinically relevant soon after treatment initiation. Accordingly, SGLT-2 inhibitors should not be regarded simply as glucose-lowering drugs with secondary cardiovascular advantages, but rather as therapies with multidomain actions across the cardiorenal–metabolic axis.

### 4.2. Hemodynamic and Renal Mechanisms

The hemodynamic profile of SGLT-2 inhibitors offers one of the clearest explanations for their cardiorenal benefit. By inhibiting proximal tubular sodium and glucose reabsorption, these agents promote natriuresis and osmotic diuresis, with a reduction in volume retention and a modest fall in systolic BP, usually in the range of about 2–5 mmHg [25,26]. In practice, however, BP lowering seems to be only one part of the story. A meta-analysis found no significant relationship between the degree of systolic BP reduction and the magnitude of CV event reduction, which argues against a purely antihypertensive explanation [27].

Their renal effects appear more central. In diabetes, enhanced proximal sodium reabsorption lowers sodium delivery to the macula densa, blunts tubuloglomerular feedback, and favors intraglomerular hypertension and hyperfiltration. SGLT-2 inhibition reverses this pattern by increasing distal solute delivery, restoring tubuloglomerular feedback, and lowering intraglomerular pressure [26,28]. This is reflected clinically by the familiar early dip in eGFR, followed by slower long-term decline in kidney function [25,28]. The downstream relevance of this renal unloading is supported by EMPEROR-Reduced, where empagliflozin slowed eGFR decline and reduced HF hospitalization and kidney events across a broad range of baseline kidney function [29].

This renal-hemodynamic model is particularly important because it links kidney protection with cardiovascular benefit in a biologically coherent way. By reducing intraglomerular stress, sodium retention, and congestion-related load, SGLT-2 inhibitors may interrupt maladaptive interactions between the kidney and the failing or vulnerable heart. Nevertheless, although these mechanisms are strongly supported by physiological and clinical observations, the relative contribution of each pathway to outcome reduction cannot be quantified with certainty and is likely to vary across patient populations.

### 4.3. Effects on Myocardial Function and HF

The benefit of SGLT-2 inhibitors in HF appears to arise from several complementary myocardial effects rather than from one isolated mechanism. These agents improve myocardial substrate handling and reduce resting myocardial oxygen consumption, pointing to a more favorable energetic profile in the failing heart. They also influence intracellular ion homeostasis and lessen ventricular loading, changes that may reduce wall stress and improve cardiac performance [30,31].

Structural improvement has been observed as well. Treatment has been associated with regression of left ventricular mass, supporting an effect on reverse remodeling. At the tissue level, SGLT-2 inhibition has been linked to lower oxidative stress, dampening of inflammatory signaling, and attenuation of profibrotic pathways, with reduced collagen-related remodeling and a lower fibrosis burden [30,32,33].

From a clinical perspective, these mechanistic changes parallel reductions in HF hospitalization and CV mortality. The HF benefit therefore fits well with the biologic profile of SGLT-2 inhibitors and helps explain why their CV action extends beyond glycaemic control alone [34].

At the same time, these myocardial mechanisms should be interpreted with appropriate caution. Some are supported primarily by experimental, imaging, or translational data rather than direct proof of causal mediation in outcome trials. The most robust clinical conclusion is therefore not that one specific myocardial pathway explains the HF benefit, but that multiple converging effects—on congestion, renal function, ventricular loading, energetics, and tissue remodeling—are biologically plausible and consistent with the observed reduction in HF events.

### 4.4. Implications for CV Prevention

SGLT-2 inhibitors now occupy a broader place in CV prevention than would be expected from their modest glucose-lowering effect alone. Contemporary guidance recommends them in people with T2D who have established ASCVD, multiple CV risk factors, diabetic kidney disease, or HF, with the aim of reducing major adverse CV events and/or HF hospitalization [35]. Their clinical importance lies in a pattern of benefit that extends across the cardiorenal continuum, particularly through consistent reductions in HF events and slowing of CKD progression, including in populations where baseline glycaemic status does not fully account for outcome differences [35,36]. The primary prevention signal appears more selective for atherosclerotic events, with benefit being more evident in individuals who have both CKD and T2D than in those with diabetes alone and no established ASCVD. This places SGLT-2 inhibitors among the therapies that have helped shift prevention from glucose-centered treatment toward integrated CV and renal risk modification [37].

From a preventive perspective, the most consistent and clinically actionable message is that SGLT-2 inhibitors are particularly well aligned with phenotypes characterized by HF risk, CKD, congestion, or combined cardiorenal vulnerability. Their effect on atherosclerotic outcomes appears more modest and less uniform than their effect on HF and kidney endpoints, and this distinction should be reflected in therapeutic positioning. In the context of the present review, this supports the broader Cardiometabolic 2.0 concept by illustrating how treatment selection can be guided not only by the presence of diabetes, but also by the dominant pattern of organ risk and anticipated clinical benefit.

## 5. GLP-1 Receptor Agonists: Mechanistic Basis for CV Protection

### 5.1. Metabolic and Weight-Reducing Effects

GLP-1 receptor agonists improve the metabolic milieu largely through reduced energy intake and sustained weight loss. In the obesity-focused semaglutide literature, appetite regulation appears central: treatment is associated with lower hunger, lower prospective food consumption, greater satiety and fullness, better control of eating, and fewer food cravings, changes that translate into lower spontaneous caloric intake. In a 20-week randomized trial, semaglutide 2.4 mg reduced ad libitum energy intake by 35% versus placebo and produced a 9.9% reduction in body weight, while indirect assessment did not support a meaningful persistent effect on overall gastric emptying at week 20, suggesting that long-term weight reduction is more plausibly driven by altered appetite and eating behavior than by sustained gastric slowing [38,39].

Beyond weight loss itself, semaglutide improves several cardiometabolic markers relevant to CV prevention. In STEP 1 and 4, reductions in waist circumference, systolic and diastolic BP, fasting plasma glucose, fasting insulin, HOMA-IR, and atherogenic lipids were greater with semaglutide than with placebo, and some participants required less antihypertensive or lipid-lowering therapy over time [40,41]. These data support the view that the CV promise of GLP-1 receptor agonists begins with a broad improvement in adiposity-related metabolic risk rather than glycaemic lowering alone. This pattern is clinically relevant because it places GLP-1 receptor agonists within a broader strategy of adiposity-related risk reduction. Their cardiovascular role is tied not simply to glucose lowering, but to improvement in the metabolic environment that drives atherosclerosis, vascular dysfunction, and long-term CV risk.

### 5.2. Vascular and Endothelial Actions

GLP-1 receptor agonists appear to support vascular homeostasis through favorable effects on endothelial signaling, oxidative stress, and vasomotor function. Endothelial dysfunction in cardiometabolic disease is closely linked to reduced nitric oxide availability, excess reactive oxygen species, impaired vasodilation, and a shift toward a pro-inflammatory, pro-atherogenic vascular phenotype. Within this context, GLP-1 receptor agonism has been associated with enhanced endothelial nitric oxide synthase activation, greater nitric oxide bioavailability, and improved endothelial-dependent vasodilation in experimental and translational settings [42,43].

The human data remain more nuanced. In obese adults with prediabetes, no clear change in flow-mediated dilation was observed in the overall cohort, which had largely preserved baseline endothelial function. In contrast, participants with lower baseline flow-mediated dilation showed improvement after treatment, suggesting that vascular benefit may be more apparent when endothelial dysfunction is already present. In the same study, liraglutide reduced monocyte chemoattractant protein-1 and plasminogen activator inhibitor-1, pointing to concurrent anti-inflammatory and profibrinolytic effects at the vascular interface [44]. Experimental evidence further indicates attenuation of oxidative stress through reduced NADPH oxidase-dependent reactive oxygen species generation, improved mitochondrial function, and restoration of endothelial redox balance, changes that fit with a broader reduction in vascular dysfunction and stiffness-related abnormalities [42,43]. However, these findings remain largely mechanistic and surrogate in nature and should not be interpreted as direct evidence that endothelial effects mediate the cardiovascular outcome benefits observed in clinical trials.

The vascular story is therefore plausible, but it should be presented with some restraint. Endothelial benefit appears more convincing in settings where vascular dysfunction is already established than in unselected populations. For that reason, the strongest clinical signal of this drug class still comes from outcome trials rather than from surrogate vascular markers alone. Accordingly, the mechanistic vascular data are best viewed as biologically supportive rather than outcome-defining.

### 5.3. Anti-Inflammatory and Anti-Atherosclerotic Effects

GLP-1 receptor agonists appear to modulate atherosclerotic disease through a broad anti-inflammatory profile that extends from circulating biomarkers to plaque-level biology. Low-grade systemic inflammation is attenuated alongside reductions in proinflammatory mediators, including TNF-α and IFN-γ, and lower circulating osteopontin has also been reported in experimental settings. In clinical analyses, semaglutide lowered hsCRP by roughly 20–30%, with the effect only partly explained by changes in body weight and glycaemic control, supporting an anti-inflammatory action that is not fully reducible to metabolic improvement alone [43,45,46]. That is of interest because it suggests that the anti-inflammatory signal cannot be attributed entirely to weight loss or improved glycaemia. At the same time, these biomarker changes should be interpreted as supportive rather than definitive, since they do not by themselves establish that anti-inflammatory effects directly mediate the cardiovascular benefit observed in outcome trials.

At the vascular wall, the inflammatory response seems to be reshaped through effects on macrophage behavior, leukocyte recruitment, and foam-cell formation. GLP-1 signaling has been linked to suppression of foam-cell formation, lower expression of adhesion-related pathways, and a shift in macrophage polarization toward an anti-inflammatory M2 phenotype, a pattern consistent with slower progression of coronary atherosclerosis [43,45,47].

These changes are mirrored by structural and imaging findings relevant to plaque stability. Experimental models have shown smaller lesions, fewer lesional leukocytes and macrophages, reduced necrotic core burden, lower MMP-9 expression, and thicker fibrous caps and greater collagen content. In advanced atherosclerosis, semaglutide also reduced PET tracer uptake reflecting activated macrophages and plaque metabolic activity, while microcalcification remained unchanged [43,45,48].

### 5.4. Implications for CV Prevention

The clinical profile of GLP-1 receptor agonists fits most naturally within prevention of atherosclerotic CV events. In people with T2D and either established CVD or CV risk factors, treatment has been associated with a lower incidence of major adverse CV events over long-term follow-up, even in populations with relatively modest baseline HbA1c values and a broad risk spectrum [49]. Current recommendations place agents with demonstrated CV benefit alongside BP, lipid, and glycaemic management as part of a core risk-reduction strategy in T2D, reflecting a treatment model that extends beyond glucose lowering alone [35]. This framework is especially relevant where obesity, diabetes, and atherosclerotic risk overlap. In adults with established CVD and overweight or obesity but without diabetes, semaglutide also reduced the incidence of CV death, nonfatal myocardial infarction, or nonfatal stroke, supporting a broader preventive role for this drug class across the cardiometabolic continuum [10]. That finding widened the clinical scope of the class and supported the view that cardiovascular benefit can extend beyond diabetes itself, particularly in patients whose risk is closely tied to excess adiposity and atherosclerotic burden. At the same time, the overall evidence is better interpreted as supporting a predominant atherosclerotic benefit pattern rather than a uniform effect across all cardiovascular domains or across all agents within the class.

**Figure 2:**
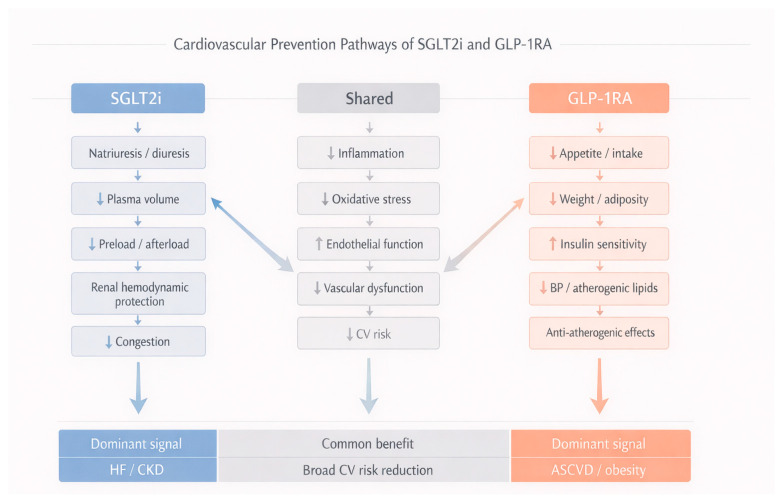
Distinct and overlapping pathways through which SGLT-2 inhibitors and GLP-1 receptor agonists contribute to cardiovascular prevention. Abbreviations: ASCVD, atherosclerotic cardiovascular disease; BP, blood pressure; CKD, chronic kidney disease; CV, cardiovascular; GLP-1RA, glucagon-like peptide-1 receptor agonists; HF, heart failure; SGLT2i, sodium-glucose cotransporter-2 inhibitors. Symbols used in the figure: ↑, increase/improvement; ↓, decrease/reduction.

Compared with SGLT-2 inhibitors, GLP-1 receptor agonists fit more naturally into a phenotype marked by obesity, residual atherosclerotic risk, and the need for meaningful weight reduction. Their effect on HF outcomes is less consistent, and their renal benefit, although promising, has generally been less pronounced than that observed with SGLT-2 inhibitors in dedicated kidney and heart failure settings. This distinction matters for therapeutic positioning and supports a phenotype-based approach to prevention across the cardiorenal–metabolic spectrum [10,49]. However, this positioning should be understood as an interpretive summary of current trial and guideline patterns rather than as a fixed comparative hierarchy. In clinical practice, overlap between obesity, ASCVD, HF, and CKD is common, and treatment selection may therefore require a broader and more individualized assessment of risk domains rather than assignment to a single therapeutic category (Figure 2).

## 6. CV Outcome Evidence

### 6.1. Major Outcome Trials of SGLT-2 Inhibitors

CV outcome evidence for SGLT-2 inhibitors first emerged in trials conducted in T2D populations at high CV risk. In EMPA-REG OUTCOME, empagliflozin reduced three-point major adverse CV events, with marked reductions in CV death, hospitalization for HF, and all-cause mortality [8]. In the CANVAS Program, canagliflozin also reduced three-point major adverse CV events and was associated with favorable renal findings, including slower progression of albuminuria and fewer renal composite events [50]. DECLARE–TIMI 58, which included a broader population of patients with established ASCVD or multiple CV risk factors, further clarified the pattern of benefit: three-point major adverse CV events were not significantly reduced, whereas the composite of CV death or hospitalization for HF was lower, driven mainly by fewer HF admissions, together with a lower rate of renal events [51]. VERTIS CV later confirmed cardiovascular safety for ertugliflozin, with neutrality for three-point major adverse CV events, fewer hospitalizations for HF, and a renal signal that did not reach statistical significance for superiority [52].

The interpretation of the class changed further when dedicated HF and CKD trials were completed. In heart failure with reduced ejection fraction (HFrEF), treatment lowered the composite of worsening HF or CV death, reduced first worsening HF events, and was associated with lower all-cause mortality. These benefits were similar in participants with and without diabetes. In CKD, treatment reduced the composite of sustained kidney function decline, end-stage kidney disease, or renal or CV death, lowered the kidney-specific composite outcome, reduced the combined risk of CV death or hospitalization for HF, and lowered all-cause mortality. Here as well, benefits were similar regardless of diabetes status [53,54].

Viewed together, these trials show a benefit profile that is more consistent for HF and renal protection than for atherosclerotic outcomes. This broader pattern was confirmed in a large collaborative meta-analysis, which found a 23% reduction in the composite of CV death or hospitalization for HF, a 37% reduction in kidney disease progression, a 23% reduction in acute kidney injury, and a modest but significant reduction in CV death, with broadly similar effects in people with and without diabetes [55].

### 6.2. Major Outcome Trials of GLP-1 Receptor Agonists

CV outcome evidence for GLP-1 receptor agonists has shown a benefit profile centered mainly on atherosclerotic events. In LEADER, liraglutide reduced three-point major adverse CV events in patients with T2D at high CV risk, with benefit driven mainly by fewer cardiovascular deaths and nonfatal myocardial infarctions [56,57]. In SUSTAIN-6, semaglutide also reduced three-point major adverse CV events, with a particularly marked effect on nonfatal stroke [58]. Harmony Outcomes showed a significant reduction in major adverse CV events with albiglutide in patients with T2D and established CVD [59], whereas REWIND extended this benefit to a broader T2D population that included many participants without established CVD at baseline [49]. By contrast, EXSCEL demonstrated cardiovascular safety for exenatide but did not show superiority for the primary composite outcome [56]. The effect was therefore not fully uniform across the class. These trials support a predominantly atherosclerotic benefit pattern, but they also show that the magnitude and composition of benefit have varied across agents, populations, and endpoints. In some studies, the benefit was driven more clearly by fewer nonfatal strokes or myocardial infarctions than by a consistent reduction in CV death [49,56,57,58,59,60].

Beyond the major adverse cardiovascular events (MACE), the signal was more selective. Hospitalization for HF was usually neutral in the diabetes CV outcome trials, without the consistent reduction observed with SGLT-2 inhibitors. Renal findings were more favorable, although they were commonly reported as secondary or exploratory outcomes and were often driven by lower rates of macroalbuminuria or slower decline in kidney function rather than by hard kidney failure endpoints. In several trials, treatment was associated with fewer new or worsening nephropathy events, and broader composite renal outcomes also improved [57,58,60]. AMPLITUDE-O added further support by showing that efpeglenatide reduced three-point major adverse CV events and was associated with improved composite renal outcomes in a population with T2D and either CVD or CKD plus a CV risk factor [60].

More direct renal outcome evidence is now available from FLOW, a dedicated kidney outcome trial in patients with T2D and CKD. Beyond confirming renal benefit, the trial also supported favorable cardiovascular and kidney-function trajectories, thereby strengthening the renal evidence base for GLP-1 receptor agonists. Nevertheless, the broader class profile remains less uniform than that of SGLT-2 inhibitors in dedicated cardiorenal settings [61].

A major extension of this evidence came from patients with established CVD and overweight or obesity without diabetes. In that setting, treatment reduced the primary composite of CV death, nonfatal myocardial infarction, or nonfatal stroke by 20%, with fewer nonfatal myocardial infarctions and a lower nephropathy composite, while CV death alone remained unchanged [10]. However, this finding should be interpreted with some caution. The follow-up remained relatively limited for a chronic prevention setting, cardiovascular death alone was not significantly reduced, and the trial does not resolve whether the observed benefit was driven predominantly by weight loss, broader cardiometabolic improvement, or multiple interacting mechanisms. Its main importance therefore lies in showing that cardiovascular benefit may extend beyond diabetes, rather than in establishing a definitive mechanism of effect [10].

Overall, this outcome pattern places GLP-1 receptor agonists primarily in the domain of atherosclerotic risk reduction, with supportive renal benefit and a weaker, less consistent HF signal [57] (Table 1).

### 6.3. Differences in Clinical Benefit Profiles

Both drug classes have moved CV prevention beyond a purely glucocentric model, but their benefit profiles differ. Current guidance supports both in T2D with established ASCVD or CKD, while their practical emphasis depends on the dominant cardiorenal–metabolic phenotype. In general, SGLT-2 inhibitors are prioritized when HF or CKD risk predominates, whereas GLP-1 receptor agonists are more closely aligned with atherosclerotic risk reduction and meaningful weight loss [35].

Outcome data broadly support this distinction. Both classes improve atherosclerotic outcomes in selected high-risk populations, but the MACE signal has generally been more consistent across positive GLP-1 receptor agonist trials than across SGLT-2 inhibitor CV outcome trials [35,61,62]. The contrast is clearer for HF and kidney endpoints: SGLT-2 inhibitors have shown more consistent reductions in HF hospitalization, CV death or HF hospitalization, and renal outcomes, particularly in dedicated HF and CKD trials, whereas GLP-1 receptor agonists have shown a weaker and less uniform HF signal while retaining favorable effects on atherosclerotic and nephropathy-related outcomes [35,62]. At the same time, these comparisons should be interpreted with caution, since they are drawn largely from separate trials enrolling different populations, using different primary endpoints, and not from direct head-to-head randomized comparisons between the two classes.

This difference should not be overstated as complete therapeutic separation. Many patients have overlapping phenotypes, and both classes may be clinically relevant in the same individual. Even so, the balance of evidence suggests that SGLT-2 inhibitors fit more naturally within HF- and kidney-centered prevention, whereas GLP-1 receptor agonists fit more naturally within prevention strategies focused on residual atherosclerotic risk, obesity, and broader metabolic risk reduction.

A direct comparison in patients with T2D and CKD adds another layer to this interpretation. In observational comparative-effectiveness analyses, initiation of an SGLT-2 inhibitor was associated with a lower risk of nonfatal myocardial infarction or stroke than initiation of a GLP-1 receptor agonist, particularly in individuals with albuminuria and preserved or near-preserved eGFR [63]. Because these findings do not come from randomized head-to-head trials, they should be interpreted cautiously. They are better understood as supportive of broader cross-trial patterns rather than as definitive comparative proof of superiority of one class over the other. In practice, however, they are consistent with the broader clinical pattern: SGLT-2 inhibitors align more closely with HF and kidney-centered prevention, whereas GLP-1 receptor agonists are often better suited when residual atherosclerotic risk and weight-related metabolic burden are the main priorities [63].

The distinct but partially overlapping benefit profiles of SGLT-2 inhibitors and GLP-1 receptor agonists are summarized in Table 2. As presented here, this comparison is intended as an interpretive clinical synthesis of the available evidence rather than as a formal hierarchy or prescriptive treatment algorithm.

### 6.4. Expansion of CV Outcome Evidence Beyond T2D

A major development in the outcome literature has been the extension of these therapies beyond T2D. Early CV outcome trials established benefit in diabetes populations, but later studies showed that the clinical value of these agents could not be explained by glucose lowering alone. Their role is now defined more clearly by the disease trajectories they modify across the cardiorenal–metabolic spectrum [53,64].

For SGLT-2 inhibitors, this expansion became most evident in HF and CKD, where benefits were observed regardless of diabetes status. This changed how the class was understood in practice. SGLT-2 inhibitors were no longer viewed mainly as glucose-lowering drugs with added CV benefit, but as therapies with direct relevance to HF progression, kidney function decline, and cardiorenal risk more broadly [53,54,64,65].

For GLP-1 receptor agonists, the extension beyond diabetes followed a different course. The clearest step came in overweight or obesity with established CVD, where semaglutide reduced major adverse CV events in the absence of diabetes [10]. This placed GLP-1 receptor agonists more firmly within a prevention model centered on obesity-related and atherosclerotic risk, and supported the view that excess adiposity itself may serve as a therapeutic entry point for CV risk reduction.

Overall, the evidence beyond T2D has helped define the different clinical domains in which these two drug classes are most relevant: SGLT-2 inhibitors mainly in HF and CKD, and GLP-1 receptor agonists mainly in obesity-associated and atherosclerotic CV risk [10,53,54,64,65].

## 7. Role in Primary and Secondary CV Prevention

### 7.1. Secondary Prevention in Established ASCVD

In secondary prevention, both drug classes have a role, but their contributions are not identical. In patients with established ASCVD, GLP-1 receptor agonists are more closely aligned with a reduction in residual atherosclerotic risk. In cardiovascular outcomes trials and secondary analyses, this has been reflected mainly in lower rates of MACE, with benefit driven largely by myocardial infarction and stroke rather than by a uniform reduction in all individual CV endpoints [66,67]. Additional reductions in coronary revascularization and selected recurrent ischemic events have also been reported in secondary prevention analyses [67]. In addition, CV benefit with semaglutide in established ASCVD was maintained regardless of baseline glycemic status or early HbA1c change, supporting a role that extends beyond glucose lowering alone [66].

SGLT-2 inhibitors also remain relevant in established ASCVD, although their profile appears more favorable when coronary disease coexists with a broader cardiorenal phenotype. In post-acute coronary settings, they were associated with lower all-cause and CV mortality, with additional reduction in recurrent myocardial infarction among patients with T2D [68]. Their place in secondary prevention is therefore often shaped less by direct anti-atherothrombotic effects than by the frequent coexistence of HF risk, CKD, or combined cardiorenal vulnerability in patients with established ASCVD.

Current recommendations therefore support either class in established ASCVD, while also recognizing their complementary value and the option of combined use when residual CV and kidney risk remain high [35,69]. In practice, GLP-1 receptor agonists are often the more natural fit when residual atherosclerotic risk predominates, whereas SGLT-2 inhibitors become especially attractive when ASCVD is accompanied by HF, CKD, or other features of cardiorenal risk.

### 7.2. Prevention in HF and CKD

In HF and CKD, SGLT-2 inhibitors are no longer best viewed as adjuncts to glycaemic management. They now occupy a central place within a cardiorenal treatment strategy. In established HF with reduced ejection fraction, treatment lowered the composite of worsening HF or CV death, reduced first worsening HF events, and was associated with lower all-cause mortality, with similar benefit in people with and without diabetes [53]. In CKD, treatment reduced the composite of kidney disease progression or CV death, lowered kidney-specific progression outcomes, and reduced hospitalization from any cause, with consistent effects across diabetes strata and across a broad range of kidney function [65].

Broader evidence supports the same clinical direction. Across HF, diabetes, and CKD populations, SGLT-2 inhibitors reduced the composite of HF hospitalization or CV death, first HF hospitalization, and CV death, with a stable effect pattern across overlapping phenotypes [70]. Recent guidance therefore places these agents among the pillars of HF prevention and treatment, with early initiation and treatment persistence encouraged in appropriate patients. This position is supported by a body of evidence that is stronger and more consistent for HF and CKD than for atherosclerotic outcomes.

GLP-1 receptor agonists may still improve overall cardiometabolic risk, but in established HF or CKD they do not occupy the same central position [71]. Their role in these settings is better understood as complementary, particularly when obesity, residual atherosclerotic risk, or the need for substantial weight reduction remains clinically important. Nevertheless, the evidence supporting GLP-1 receptor agonists in HF and CKD remains less uniform than that supporting SGLT-2 inhibitors in dedicated cardiorenal settings. For this reason, their role in these phenotypes is better viewed as complementary and context-dependent rather than equivalent. This distinction reflects the current balance of evidence and should not be interpreted as a rigid treatment hierarchy.

### 7.3. Primary Prevention in High-Risk Patients

In primary prevention, treatment choice is less straightforward because risk is distributed across several overlapping phenotypes, including T2D without overt CVD, obesity, early kidney vulnerability, and clustered metabolic risk factors. In people with T2D who had no prior macrovascular or major microvascular disease, SGLT-2 inhibitor use was associated with lower risks of incident coronary artery disease, HF, CV death, dialysis, vision-threatening retinopathy, amputation, and all-cause mortality, supporting a role that extends beyond glycemic control toward early vascular and renal protection [72].

GLP-1 receptor agonist data also point to benefit before overt CVD is established. In a broad-risk diabetes population, treatment reduced the burden of total major CV or fatal events and also lowered expanded event burden, including unstable angina, HF, or revascularization [73]. In overweight or obesity without diabetes, CV event reduction was maintained across baseline HbA1c strata and across early HbA1c change categories, showing that benefit can emerge even before diabetes is present and is not dependent on baseline dysglycemia alone [66].

At the same time, primary prevention should not be framed as a uniform indication for either class. The more appropriate interpretation is that benefit becomes more compelling as risk begins to cluster around a dominant phenotype, such as early CKD or HF vulnerability for SGLT-2 inhibitors, or obesity with elevated atherosclerotic risk for GLP-1 receptor agonists. This fits well with the CKM framework, which views obesity, dysglycemia, kidney dysfunction, and vascular risk as part of a shared disease trajectory and supports earlier screening and phenotype-based intervention [11]. Current recommendations similarly place agents with proven CV and kidney benefit among the core components of multifactorial risk reduction in T2D [35].

### 7.4. Earlier Intervention and Residual Risk Reduction

CV prevention is moving away from a late, event-driven model toward earlier treatment along the cardiometabolic disease trajectory. A phenotype-prioritized approach is increasingly favored, in which therapy is aligned with the dominant pattern of risk—atherosclerotic, HF, CKD, obesity, or overlapping multimorbidity—rather than selected mainly according to glycaemic status. In this setting, SGLT-2 inhibitors and GLP-1 receptor agonists are viewed as organ-protective therapies, and combination or sequencing strategies are being considered in patients whose CV and renal risk remains high despite standard care [74,75].

This earlier perspective is supported by longitudinal data showing that progression in CKM stage is associated with a higher risk of death, HF, stroke, and myocardial infarction, with particularly unfavorable patterns in older adults and in women [76]. Serial staging may help identify patients whose risk is rising before overt events occur and who may benefit from earlier intensification of preventive therapy.

At the same time, residual risk is not explained by biology alone. Real-world data show that uptake of organ-protective therapies has improved, yet many eligible patients remain untreated, combination therapy is still uncommon, and care gaps persist in groups such as women and patients with CKD [75,77]. Implementation, follow-up, and multidisciplinary coordination should therefore be regarded as part of prevention itself rather than as issues separate from treatment efficacy.

## 8. Guideline Integration and Clinical Positioning

### 8.1. Convergence of Cardiology, Diabetology, and Nephrology Recommendations

Recent recommendations from diabetology, cardiology, and nephrology are moving in the same direction. SGLT-2 inhibitors and GLP-1 receptor agonists are no longer positioned chiefly as glucose-lowering therapies. They are now incorporated into risk reduction strategies that address atherosclerotic CVD, HF, CKD, and, increasingly, obesity-related cardiometabolic disease [6,35,78].

Diabetology guidance now places these agents within the core framework of CV and kidney risk reduction in T2D, alongside BP, lipid, and glycaemic management, rather than reserving them for later intensification of glucose-lowering therapy [35]. Cardiology guidance has also moved beyond an HbA1c-centered approach, integrating these therapies into broader CV management and recognizing that their clinical value differs according to the presence of ASCVD, HF, CKD, and weight-related risk [6]. Nephrology recommendations have evolved in parallel, placing kidney protection and cardiorenal risk reduction at the center of CKD care rather than treating renal outcomes in isolation [78].

This convergence is well captured by the CKM framework, which links adiposity, dysglycaemia, kidney dysfunction, and CVD within a shared continuum and supports earlier screening, staging, and intervention across the life course [11]. It also helps explain why treatment decisions increasingly depend on the dominant phenotype and on coordinated care across specialties, rather than remaining confined within traditional disciplinary boundaries [75].

### 8.2. Patient Phenotypes and Treatment Prioritization

Current positioning of these therapies is increasingly phenotype-based rather than glucose-centered. When residual atherosclerotic risk predominates, GLP-1 receptor agonists may often be favored because they are associated with reductions in major adverse CV events, myocardial infarction, and stroke, with added relevance in patients with higher BMI, where weight reduction may further modify residual ASCVD risk [67].

By contrast, when HF, CKD, or broader cardiorenal vulnerability define the clinical picture, SGLT-2 inhibitors may be particularly relevant. Their profile is centered on lower HF hospitalization, slower renal functional decline, and benefit that extends beyond diabetes, which places them in a central position within the cardiorenal axis [79].

This distinction becomes more relevant in obesity and early cardiometabolic multimorbidity, where excess adiposity, insulin resistance, inflammation, and overlapping organ risk often coexist. In such settings, a phenotype-based approach may support preferential consideration of GLP-1 receptor agonists when weight reduction and atherosclerotic risk modification are the main goals, and SGLT-2 inhibitors when HF or kidney protection is the more pressing concern [74].

Recent standards also support individualized selection outside a rigid metformin-first sequence. In T2D with established ASCVD, high ASCVD risk, HF, or CKD, an SGLT-2 inhibitor and/or GLP-1 receptor agonist with proven benefit may be chosen according to the dominant phenotype, and combined or sequential use may be reasonable when risk domains overlap [80]. At the same time, this type of positioning should not be interpreted as a fixed hierarchy or as a substitute for individualized guideline-based care. It reflects a practical clinical synthesis derived primarily from randomized trials and guideline documents, but also from broader interpretive reasoning in areas where direct comparative evidence remains limited. Because this type of positioning draws partly on trial evidence and partly on clinical interpretation, it is better understood as a practical treatment framework than as a formal hierarchy. Table 3 summarizes an author-derived phenotype-based approach to the positioning of SGLT-2 inhibitors and GLP-1 receptor agonists in CV prevention. The table is intended as a didactic aid to phenotype-aware clinical reasoning rather than as a guideline-endorsed algorithm or a prescriptive treatment sequence.

### 8.3. Implications for Clinical Practice

Clinical practice is moving toward prevention algorithms that are increasingly cardiometabolic rather than narrowly glucose-centered. Current standards support the use of SGLT-2 inhibitors and GLP-1 receptor agonists according to CV, renal, and weight-related risk, and allow these agents to be introduced in selected patients without waiting for a rigid escalation sequence based solely on HbA1c or background metformin therapy [80].

This shift has practical consequences. The clinical question is no longer confined to glycaemic correction, but extends to prevention of HF, kidney disease progression, atherosclerotic events, and excess adiposity-related risk. Recent integrative reviews describe care models in which cardiology, diabetology, nephrology, primary care, pharmacy, and nutrition work within shared pathways or dedicated cardiometabolic clinics, allowing treatment to be aligned more closely with the patient’s dominant phenotype and residual risk profile [14,81].

At the same time, implementation remains uneven. In contemporary primary care, these agents are still underused in guideline-eligible patients, with lower use in older adults, marked variation across centers, and substantial cost implications if uptake is expanded. Prescribing disparities and fragmented care pathways therefore remain major barriers to translating evidence into routine prevention [14,82].

## 9. Practical Challenges and Unresolved Questions

### 9.1. Access, Cost, and Reimbursement

Despite strong guideline support, use of these agents remains well below what eligibility would justify in routine care. In a contemporary primary care setting, dispensing rates remained modest, declined with age, varied markedly across centers, and, for SGLT-2 inhibitors, were lower in women, pointing to uneven implementation even within a publicly subsidized system [82]. In another high-risk primary care population, most eligible individuals still did not receive either class, showing that the gap between recommendations and prescribing persists even after guideline updates [83].

Cost remains a major barrier. Higher out-of-pocket costs were associated with lower initiation of both classes and with longer delays before treatment intensification, indicating that affordability directly shapes access [84]. Broader health-system analyses add that underuse is also driven by fragmented care, reimbursement limitations, clinical inertia, and social determinants of health, with the heaviest burden often falling on those already at highest cardiometabolic risk [85]. In practice, these barriers mean that patients most likely to benefit from organ-protective therapy are not always the ones most likely to receive it.

### 9.2. Adherence, Tolerability, and Safety

Tolerability remains a major determinant of treatment persistence in routine care. For GLP-1 receptor agonists, gastrointestinal adverse effects are the main limitation, particularly nausea, vomiting, diarrhea, and constipation, most often during early treatment and after dose escalation, with a clear dose-dependent pattern. Delayed gastric emptying has also gained attention in procedural settings because retained gastric contents may persist despite temporary discontinuation, which has led to symptom-based perioperative assessment and, in selected cases, gastric ultrasound or treatment interruption before higher-risk procedures. Hepatobiliary events, especially gallbladder and biliary disease, also warrant attention, while pancreatitis remains an area that calls for clinical vigilance rather than a settled causal interpretation [86,87].

For SGLT-2 inhibitors, the most frequent adverse effects are genital mycotic infections and volume depletion, which are usually mild and manageable. Euglycemic ketoacidosis is uncommon but clinically important, particularly during fasting, acute illness, or the perioperative period. For this reason, routine practice now includes patient education on sick-day management, attention to hydration and BP, and temporary discontinuation before surgery or prolonged fasting [88,89].

These issues have a direct effect on adherence. Within 1 year, discontinuation affected 23.6% of GLP-1 receptor agonist users and 27.9% of SGLT-2 inhibitor users; by 3 years, these proportions rose to 38.5% and 45.9%, respectively. At the same time, reinitiation was frequent, and overall treatment coverage remained around 70–80% over 1–5 years, indicating that persistence is better understood as a dynamic pattern of use than as a simple yes-or-no measure [89].

### 9.3. Combination Therapy and Sequencing

Questions around sequencing remain open because current practice has moved faster than the evidence base. Available data support the biological plausibility of combining these two classes, particularly in patients with overlapping CV, renal, and metabolic risk, but direct comparative evidence showing the best order of initiation is still limited. Most published studies are observational, heterogeneous in design, and focused more on surrogate outcomes than on hard long-term cardiorenal endpoints [90]. In practice, the first agent is usually chosen according to the dominant phenotype. When HF or CKD is the main concern, SGLT-2 inhibitors often move forward first because of their stronger cardiorenal positioning. When residual atherosclerotic risk, obesity, or the need for greater weight reduction predominates, GLP-1 receptor agonists may be the more attractive starting point. In patients with persistent albuminuria, declining kidney function, or residual CV risk despite one class, adding the other is increasingly viewed as a reasonable next step rather than a late rescue strategy [91,92].

At present, sequential intensification remains more common than simultaneous initiation, shaped not only by phenotype and treatment goals, but also by tolerability, adherence, cost, and reimbursement. The rationale for combination therapy is clinically compelling, but the evidence base for optimal sequencing remains incomplete [90,91].

### 9.4. Remaining Evidence Gaps

Several questions remain open as these therapies move earlier and more broadly across the cardiometabolic spectrum. Evidence is strongest in patients at high cardiorenal risk, whereas lower-risk populations remain less clearly defined. In earlier-stage disease, available data suggest favorable effects on body weight, fasting glucose, and glycaemic measures, with possible delay in progression to overt diabetes, but the evidence remains limited and is not yet sufficient to define a clear preventive role in routine prediabetes care [93].

Uncertainty also persists beyond diabetes. In HF, GLP-1 receptor agonists appear more promising in obesity-related phenotypes with preserved ejection fraction, where improvements in symptoms, functional capacity, and weight have been reported. By contrast, reductions in hospitalization or mortality have not been shown consistently, and their role in reduced ejection fraction remains unsettled [94].

Comparative effectiveness in phenotype-defined groups also requires better resolution. In CKD, one class was associated with a lower risk of nonfatal myocardial infarction or stroke than the other, but this evidence comes from observational data and cannot replace direct head-to-head randomized comparisons [63]. Long-term combination strategies face similar limits. Although benefit appears plausible and is increasingly explored in high-risk settings, data remain sparse for lower-risk populations, non-diabetic CKD, long-term safety, and cost-effective implementation in broader real-world care [95].

## 10. Future Directions

The next phase of CV prevention is moving toward earlier, more individualized, and more integrated care. Treatment models based mainly on glucose thresholds or single-organ disease are gradually being replaced by approaches that align therapy with phenotype, complication burden, and the broader biology of cardiometabolic dysfunction. In obesity care, this means looking beyond body weight alone and considering metabolic, CV, renal, hepatic, mechanical, and behavioral features, together with age and life stage [96].

This perspective also supports earlier intervention in obesity and preclinical disease. A newer diagnostic framework distinguishes between clinical obesity, in which excess adiposity is already associated with measurable functional impairment, and preclinical obesity, in which organ function is still preserved but risk is rising. For prevention, this distinction is relevant because it identifies a stage at which intervention may begin before overt CVD, HF, or CKD is established. It also supports a broader assessment of adiposity distribution, functional markers, and overall risk trajectory rather than reliance on BMI alone [97].

Combination strategies are also likely to become more relevant as overlapping phenotypes become increasingly common. SGLT-2 inhibitors and GLP-1 receptor agonists are already being used within broader organ-protective strategies, and newer incretin-based approaches may further expand this landscape [98]. The main clinical question is likely to shift from whether these therapies should be used in isolation to how they should be combined, sequenced, and targeted across different residual-risk domains. This is especially relevant in patients with concurrent obesity, CKD, HF, and atherosclerotic disease, where single-class treatment may leave substantial risk untreated.

Another area of development is the integration of biomarkers, imaging, and digital medicine into prevention pathways. Recent reviews describe a growing role for molecular, biochemical, imaging, physiological, and anthropometric markers in both primary and secondary prevention, with increasing interest in artificial intelligence (AI) as a tool for improving risk discrimination and treatment personalization [99]. Digital approaches, including AI-assisted imaging, wearable monitoring, telemedicine, and data-driven risk profiling, may support earlier recognition of subclinical disease and more responsive treatment adjustment over time. At the same time, their clinical value will depend on validation, accessibility, interoperability, and appropriate ethical oversight [100].

Over time, these developments may further support dedicated cardiorenal–metabolic prevention models. Rather than managing CV, renal, hepatic, and metabolic disease in separate clinical silos, future care is more likely to rely on shared frameworks, multidisciplinary teams, and earlier deployment of organ-protective therapies [98,100]. The broader direction is not simply toward more precise risk prediction, but toward care systems that can translate that precision into routine practice.

## 11. Conclusions

CV prevention is changing as the limitations of a purely glucose-centered model have become increasingly clear. As discussed in this review, SGLT-2 inhibitors and GLP-1 receptor agonists have moved beyond their original role as glucose-lowering drugs and are now positioned within broader strategies aimed at reducing cardiovascular, renal, and metabolic risk across the cardiorenal–metabolic continuum.

Their clinical profiles are distinct but complementary. SGLT-2 inhibitors are more consistently associated with protection against HF, progression of CKD, and cardiorenal events, whereas GLP-1 receptor agonists are more closely aligned with reduction in atherosclerotic events, body weight, and residual metabolic risk. However, these patterns should be interpreted as prevailing clinical tendencies rather than as absolute or universally exclusive treatment domains, particularly given the frequent overlap of ASCVD, HF, CKD, obesity, and T2D in routine practice. The extension of benefit beyond T2D, including HF and CKD for SGLT-2 inhibitors and obesity-associated CV risk for GLP-1 receptor agonists, has further clarified that these classes are best understood within a broader organ-protective framework rather than within diabetology alone.

This change has practical implications for prevention. Clinical decision-making is increasingly guided by dominant risk domains such as ASCVD, HF, CKD, and obesity, with growing emphasis on earlier intervention and coordination across cardiology, diabetology, nephrology, obesity medicine, and primary care. Nevertheless, the degree to which phenotype-based treatment positioning can be operationalized remains partly dependent on indirect evidence, evolving guideline interpretation, and clinical judgment, rather than on universally validated head-to-head treatment hierarchies. At the same time, persistent implementation gaps, cost barriers, and uneven uptake continue to limit the full translation of trial evidence into routine care.

In this context, Cardiometabolic 2.0 may be understood as a practical interpretive framework for contemporary prevention. As used in this review, it does not replace established CKM or cardiorenal–metabolic models, but highlights a treatment approach centered on organ protection, residual risk reduction, and phenotype-informed therapy across overlapping clinical domains. From this perspective, SGLT-2 inhibitors and GLP-1 receptor agonists may be regarded as major components of contemporary cardiovascular prevention within an increasingly integrated cardiorenal–metabolic model of care, while acknowledging that some aspects of comparative positioning remain more interpretive than definitive.

## Figures and Tables

**Table 1:** Landmark CV outcome trials of SGLT-2 inhibitors and GLP-1 receptor agonists.

Drug/Trial	Population	Sample Size	Follow-Up	Primary Endpoint	MACE	HHF	CV Death	Renal Outcome	Key Takeaway
**SGLT-2 inhibitors**									
Dapagliflozin/DAPA-HF [53]	Symptomatic HFrEF, with or without T2D	4744	18.2 months	Worsening HF or CV death	Not a dedicated MACE trial	Reduced, HR 0.70	Reduced, HR 0.82	Not significant, HR 0.71	Established benefit in HFrEF
Ertugliflozin/VERTIS CV [52]	T2D with established ASCVD	8246	3.5 years	3-point MACE	Noninferior; no superiority, HR 0.97	Reduced, HR 0.70	Not significant, HR 0.92	No significant renal superiority shown, HR 0.81	CV safety with stronger HF than MACE signal
Dapagliflozin/DAPA-CKD [54]	CKD, with or without T2D	4304	2.4 years	Sustained eGFR decline, ESKD, or renal/CV death	Not a dedicated MACE trial	Reduced composite of CV death or HHF, HR 0.71	Not significant, HR 0.81	Reduced, HR 0.61	Dedicated renal and cardiorenal benefit in CKD
Empagliflozin/EMPA-REG OUTCOME [8]	T2D with established CVD	7020	3.1 years	3-point MACE	Reduced, HR 0.86	Reduced, HR 0.65	Reduced, HR 0.62	Not a dedicated renal outcome trial	First SGLT-2 inhibitor trial to show MACE superiority
Canagliflozin/CANVAS Program [50]	T2D with established CVD or high CV risk	10,142	188.2 weeks	3-point MACE	Reduced, HR 0.86	Reduced, HR 0.67	Not significant, HR 0.87	Favorable exploratory renal signal, HR 0.60	MACE benefit with HF and renal signal
Dapagliflozin/DECLARE–TIMI 58 [51]	T2D with ASCVD or multiple risk factors	17,160	4.2 years	MACE; CV death or HHF	Not significant, HR 0.93	Reduced, HR 0.73	Not significant, HR 0.98	Favorable renal signal, HR 0.76	Stronger HF and renal than MACE signal
**GLP-1 receptor agonists**									
Efpeglenatide/AMPLITUDE-O [60]	T2D with CVD, or CKD plus a CV risk factor	4076	1.81 years	3-point MACE	Reduced, HR 0.73	Reduced, HR 0.61	Not significant, HR 0.72	Favorable composite renal outcome, HR 0.68	MACE benefit with supportive renal signal
Semaglutide/SELECT [10]	Overweight or obesity with established CVD, without diabetes	17,604	39.8 months	3-point MACE	Reduced, HR 0.80	HF composite reduced, HR 0.82 *	Not significant, HR 0.85	Reduced, HR 0.78	Extended CV benefit beyond diabetes
Albiglutide/Harmony Outcomes [59]	T2D with established CVD	9463	1.6 years	3-point MACE	Reduced, HR 0.78	Not significant, HR 0.85 *	Not significant, HR 0.93	Not a dedicated renal outcome trial	Atherosclerotic benefit without clear HF effect
Semaglutide/SUSTAIN-6 [58]	T2D at high CV risk	3297	2.1 years	3-point MACE	Reduced, HR 0.74	Not significant, HR 1.11	Not significant, HR 0.98	Favorable exploratory renal signal, HR 0.64	MACE benefit, particularly for stroke
Exenatide/EXSCEL [56]	T2D with or without prior CVD	14,752	3.2 years	3-point MACE	Noninferior; no superiority, HR 0.91	Not significant, HR 0.94	Not significant, HR 0.88	Not a dedicated renal outcome trial	CV safety without superiority for MACE
Dulaglutide/REWIND [49]	T2D with prior CVD or CV risk factors	9901	5.4 years	3-point MACE	Reduced, HR 0.88	Not significant, HR 0.93	Not significant, HR 0.91	Favorable composite renal outcome, HR 0.85	Benefit extended to a broader T2D population
Semaglutide/FLOW [61]	T2D with CKD	3533	3.4 years	Major kidney disease events	Reduced, HR 0.82	Not a dedicated HHF trial	Reduced, HR 0.71	Reduced primary kidney outcome, HR 0.76; kidney-specific composite HR 0.79	Dedicated kidney outcome evidence supporting renal and CV benefit in T2D with CKD

Abbreviations: ASCVD, atherosclerotic cardiovascular disease; CKD, chronic kidney disease; CV, cardiovascular; CVD, cardiovascular disease; eGFR, estimated glomerular filtration rate; ESKD, end-stage kidney disease; GLP-1, glucagon-like peptide-1; HF, heart failure; HFrEF, heart failure with reduced ejection fraction; HHF, hospitalization for heart failure; HR, hazard ratio; MACE, major adverse cardiovascular events; SGLT-2, sodium-glucose cotransporter-2; T2D, type 2 diabetes. * The heart failure composite endpoint included death from cardiovascular causes, hospitalization for heart failure, or an urgent medical visit for heart failure. Because this was a composite endpoint and not isolated hospitalization for heart failure, it should not be interpreted as directly equivalent to the dedicated HF signals reported in SGLT-2 inhibitor trials.

**Table 2:** Comparative Cardiometabolic Profile of SGLT-2 Inhibitors versus GLP-1 Receptor Agonists.

Feature	SGLT-2 Inhibitors	GLP-1 Receptor Agonists	Predominant Clinical Implication
Glycaemic effect	Moderate glucose lowering	Moderate to strong glucose lowering	Both improve glycaemic control, although GLP-1 receptor agonists often produce greater HbA1c reduction
Weight reduction	Modest	Greater and more consistent	GLP-1 receptor agonists may be particularly relevant when weight reduction is a major treatment goal
MACE	Favorable, but less uniform across CV outcome trials	More consistent reduction across positive CV outcome trials	GLP-1 receptor agonists are often more closely aligned with atherosclerotic risk reduction
Hospitalization for HF	Clear and consistent reduction	Usually neutral or modest effect in diabetes CV outcome trials	SGLT-2 inhibitors are often preferred when HF prevention is a priority
CV death	Reduced in selected trials, particularly in HF- and cardiorenal-oriented settings	Less consistent reduction as an isolated endpoint	CV death benefit appears more consistently supported for SGLT-2 inhibitors in cardiorenal settings
Renal protection	Strong and consistent, including slowing of kidney function decline and benefit in CKD	Favorable renal signal, often driven by albuminuria or composite renal outcomes	SGLT-2 inhibitors are generally preferred when kidney protection is a central objective
BP effect	Modest reduction, partly related to natriuresis and osmotic diuresis	Modest reduction	Both improve BP modestly, but this is not the main differentiating feature
Atherosclerotic risk reduction	Present, although less dominant than HF and renal effects	More prominent feature of the class	GLP-1 receptor agonists may be particularly relevant when ASCVD risk is the dominant concern
HF benefit	Major class strength	Secondary or inconsistent	SGLT-2 inhibitors fit better in HF-prone phenotypes
CKD benefit	Strong across diabetic and non-diabetic CKD settings	Supportive, but less established for hard kidney endpoints	SGLT-2 inhibitors currently have the more established CKD-focused evidence base
Evidence beyond T2D	Strong in HF and CKD populations without diabetes	Strong in overweight or obesity with established CVD without diabetes	Both classes extend beyond diabetes, but into different clinical domains
Best-fit clinical phenotype *	Patient with HF, CKD, volume-sensitive hypertension, or combined cardiorenal risk	Patient with obesity, high residual atherosclerotic risk, need for weight reduction, or broader metabolic risk reduction	Treatment choice should follow the dominant cardiorenal–metabolic phenotype rather than glucose lowering alone

* This row represents an author-derived clinical synthesis based on the evidence discussed in Section 6.1, Section 6.2 and Section 6.3 and is intended as an interpretive summary rather than a formal guideline classification. Source: Based on the comparative evidence discussed in Section 6.3 and supported by the landmark CV outcome trials summarized in Section 6.1 and Section 6.2. Abbreviations: ASCVD, atherosclerotic cardiovascular disease; BP, blood pressure; CKD, chronic kidney disease; CV, cardiovascular; CVD, cardiovascular disease; GLP-1, glucagon-like peptide-1; HbA1c, glycated hemoglobin; HF, heart failure; MACE, major adverse cardiovascular events; SGLT-2, sodium-glucose cotransporter-2; T2D, type 2 diabetes.

**Table 3:** Practical Phenotype-Based Positioning in CV Prevention.

Predominant Phenotype	Class Often Prioritized	Main Rationale	Typical Clinical Context	When to Consider the Other Class or Combined/Sequential Use
Established ASCVD/residual atherosclerotic risk	GLP-1 receptor agonist	More closely aligned with reduction in atherosclerotic events, particularly myocardial infarction and stroke, with additional benefit on weight and metabolic risk	Prior myocardial infarction, ischemic stroke, symptomatic peripheral arterial disease, persistent ASCVD risk despite standard preventive therapy	Consider SGLT-2 inhibitor when HF, CKD, or broader cardiorenal vulnerability is also present
HF/cardiorenal vulnerability	SGLT-2 inhibitor	More consistent benefit on HF hospitalization, CV death, and cardiorenal protection	HFrEF or HFpEF, congestion-prone phenotype, recurrent HF events, diabetes with emerging cardiorenal risk	Consider GLP-1 receptor agonist when obesity, residual ASCVD risk, or unmet weight-related goals remain prominent
CKD	SGLT-2 inhibitor	Slows kidney function decline and lowers cardiorenal event risk; central role in organ protection	Albuminuric CKD, declining eGFR, diabetes with renal involvement, CKD with HF risk	Consider GLP-1 receptor agonist when ASCVD or obesity is prominent, or when additional metabolic and weight benefit is desired
Obesity/excess adiposity/early CKM state	GLP-1 receptor agonist	Greater effect on body weight, adiposity-related risk, and atherosclerotic-metabolic burden	Overweight or obesity, visceral adiposity, insulin resistance, early CKM-stage disease, high-risk patients without overt CVD	Consider SGLT-2 inhibitor when HF risk, CKD, albuminuria, or cardiorenal vulnerability emerges
T2D without overt CVD but with high CV risk	Phenotype-dependent	Selection is guided by the dominant risk domain rather than by HbA1c alone	Multiple risk factors, subclinical organ damage, long diabetes duration, clustered metabolic abnormalities	Consider GLP-1 receptor agonist if ASCVD or obesity risk predominates; consider SGLT-2 inhibitor if HF or CKD risk predominates
Overlapping phenotypes (e.g., ASCVD + HF, ASCVD + CKD, obesity + CKD)	Combined or sequential use may be reasonable	Different classes address different residual-risk domains; overlapping phenotypes often require broader organ protection	Multimorbidity, persistent residual risk despite standard care, incomplete control with a single class	The first agent may be chosen according to the most clinically pressing phenotype, with later intensification based on residual risk, tolerability, kidney function, and weight goals

Abbreviations: ASCVD, atherosclerotic cardiovascular disease; CKD, chronic kidney disease; CKM, cardiovascular–kidney–metabolic; eGFR, estimated glomerular filtration rate; GLP-1, glucagon-like peptide-1; HFpEF, heart failure with preserved ejection fraction; HFrEF, heart failure with reduced ejection fraction; SGLT-2, sodium-glucose cotransporter-2. This table represents an author-derived interpretive synthesis intended to support phenotype-aware clinical reasoning. It does not replace guideline recommendations, individualized clinical judgment, or drug-specific contraindications and practical considerations.

## Data Availability

No new data were created or analyzed in this study. Data sharing is not applicable to this article.

## Supplementary Materials

The following supporting information can be downloaded at: https://www.mdpi.com/article/10.3390/life16050756/s1. Table S1. Structured search framework used for this narrative review.

## Abbreviations

The following abbreviations are used in this manuscript:

ASCVD	Atherosclerotic cardiovascular disease
BMI	Body mass index
BP	Blood pressure
CKD	Chronic kidney disease
CKM	Cardiovascular–kidney–metabolic
CV	Cardiovascular
CVD	Cardiovascular disease
DALYs	Disability-adjusted life-years
eGFR	Estimated glomerular filtration rate
ESKD	End-stage kidney disease
GLP-1 receptor agonists	Glucagon-like peptide-1 receptor agonists
HbA1c	Glycated hemoglobin
HFrEF	Heart failure with reduced ejection fraction
HHF	Hospitalization for heart failure
HOMA-IR	Homeostasis model assessment of insulin resistance
HR	Hazard ratio
hsCRP	High-sensitivity C-reactive protein
IFN-γ	Interferon gamma
MACE	Major adverse cardiovascular events
MMP-9	Matrix metalloproteinase-9
NADPH	Nicotinamide adenine dinucleotide phosphate
PET	Positron emission tomography
SGLT-2 inhibitors	Sodium-glucose cotransporter-2 inhibitors
STEP	Semaglutide Treatment Effect in People with obesity
T2D	Type 2 diabetes
TNF-α	Tumor necrosis factor alpha
