# Supplementary material for: Cardiometabolic 2.0: Redefining Cardiovascular Prevention Through SGLT-2 Inhibitors and GLP-1 Receptor Agonists
Source: Life (Basel). 2026 May 1;16(5):756. doi: 10.3390/life16050756 (PMC13208886; doi:10.3390/life16050756)
Supplement: Supplementary file 1 [file life-16-00756-s001.zip › life-4250554-supplementary.pdf]

Supplementary Table S1. Structured search framework used for this narrative review.

Item	Description
Review type	Narrative review with structured literature search
Objective	To synthesize the mechanistic rationale, outcome evidence, guideline evolution, and clinical positioning of SGLT-2 inhibitors and GLP-1 receptor agonists in cardiovascular prevention across the cardiorenal-metabolic continuum
Databases	PubMed/MEDLINE, Scopus, Web of Science
Search period	January 2019 to March 2026
Earlier studies	Landmark pre-2019 studies were included selectively when essential for understanding cardiovascular outcome-based therapy and the development of cardiorenal-metabolic treatment frameworks
Main domains searched	Cardiovascular prevention, atherosclerotic cardiovascular disease, heart failure, chronic kidney disease, obesity, type 2 diabetes, cardiorenal-metabolic continuum, mechanisms, guidelines
Interventions of interest	SGLT-2 inhibitors and GLP-1 receptor agonists
Representative search terms	"SGLT-2 inhibitors", "GLP-1 receptor agonists", "cardiovascular prevention", "cardiorenal-metabolic", "atherosclerotic cardiovascular disease", "heart failure", "chronic kidney disease", "obesity", "type 2 diabetes", "cardiovascular outcome trials", "guidelines", "mechanisms"
Representative Boolean logic	Intervention terms were combined with disease-domain and evidence-type terms using Boolean operators (AND/OR), with syntax adapted to each database
Additional search strategy	Reference lists of key trials, meta-analyses, guidelines, scientific statements, and major reviews were screened manually
Evidence prioritized	Randomized cardiovascular, heart failure, kidney, and obesity outcome trials; meta-analyses; evidence-based guidelines; scientific statements; expert consensus documents
Additional evidence included	Mechanistic, translational, and imaging studies; selected high-quality narrative or state-of-the-art reviews for contextual interpretation
Evidence prioritization logic	Priority was given to clinically influential randomized trials and meta-analyses, followed by current guidelines and consensus documents, then mechanistic and translational studies
Inclusion principles	Studies were selected based on clinical relevance, methodological robustness, recency, and direct relevance to the manuscript's central theme
Exclusion principles	Case reports, brief opinion pieces, low-informative commentaries, duplicate reports without added value, and superseded interim analyses were generally excluded
Language	English-language peer-reviewed literature was preferentially included
Review registration	No protocol was prospectively registered
Risk-of-bias assessment	No formal risk-of-bias tool was applied because the article was designed as a narrative review

Screening process	No PRISMA flow diagram or formal dual-reviewer screening process was used
Main limitation	As a narrative review, it remains subject to selection bias and interpretive bias
Interpretive safeguard	Clinical statements were revised to distinguish guideline-supported recommendations from author-derived interpretation and to avoid overstatement in the absence of head-to-head randomized evidence
